# Neutrophil to lymphocyte ratio (NLR) and YKL-40 as potential markers for discriminating mycoplasma pneumoniae pneumonia from viral pneumonia in children

**DOI:** 10.1515/med-2025-1335

**Published:** 2026-02-10

**Authors:** Yu Wan, Jun Lv, Fei Jiang, Fei Fan

**Affiliations:** Department of Pediatrics, The Second People’s Hospital of Changzhou, The Third Affiliated Hospital of Nanjing Medical University, Changzhou, China

**Keywords:** mycoplasma pneumoniae pneumonia, viral pneumonia, YKL-40, interleukin‐37, NLR

## Abstract

**Objectives:**

Our research aimed to explore the potential indicators in discriminating mycoplasma pneumoniae pneumonia (MPP) from viral pneumonia (VP) in children.

**Methods:**

TaqMan PCR testing was used to detect respiratory pathogens in all pneumonia patients. The serum levels of IL-37 and YKL-40 were measured by ELISA. While the expression levels of YKL-40 and IL37 mRNA in PBMCs were detected by qRT-PCR. We calculated the neutrophil to lymphocyte ratio (NLR) and platelet to lymphocyte ratio (PLR) values from blood routine test results. This study had been registered in the Chinese Clinical Trials Registry System (MR-32-24-025608).

**Results:**

170 patients were selected, including 85 MPP patients and 85 VP patients. In addition, 85 healthy control children were selected for comparison. The levels of serum YKL-40, IL-37 and their mRNA expressions in MPP patients were significantly higher than in VP patients and healthy control. Similarly, MPP patients had the highest levels of NLR and PLR. According to the ROC curve result, the combination of two indicators (YKL-40 and NLR) was the strongest predictor of MP vs. VP.

**Conclusions:**

These results indicate that YKL-40 and NLR constitute the critical combination of biomarkers useful for differentiating between MPP and VP in children.

## Introduction

*Mycoplasma pneumoniae* (MP) is one of the main pathogens in community-acquired pneumonia (CAP) children. In several countries that have included the pneumococcal conjugate vaccine (13-valent) in their national vaccination plans, MP has presented as the primary cause of childhood CAP [1]. Clinical signs of MP infection are often minor and self-limiting. However, there have been cases that Mycoplasma pneumonia can be fatal, causing lung necrosis and acute respiratory distress syndrome [2], 3]. Children infected with MP are older than other CAP patients and have longer duration of fever and cough symptoms. If exact antibiotic treatment is initiated in the early stages of pneumonia, the time for symptoms and signs to appear will be shortened. MP, virus, and bacteria are all major pathogens of CAP. MP and bacterial infections can be differentiated by some laboratory indicators such as white blood cell and C-reactive protein. Nevertheless, *M. pneumoniae* pneumonia (MPP) and viral pneumonia (VP) cannot be distinguished in clinical symptoms. *M. pneumoniae* pneumonia (MPP) in children also cannot be accurately diagnosed based on clinical manifestations. Due to lack of suitable testing equipment, some primary care centers, such as community hospitals frequently miss the diagnosis of MP. A recent study had found that MP CAP may be mistakenly identified as a viral infection when using novel biomarkers included in the BV score [4]. Therefore, we are trying to find some inflammatory markers that can distinguish MPP from VP in the early phase.

Recent researches showed that MP infection in the lower respiratory tract leaded to the release of inflammatory cytokines from epithelial cells and macrophages [5], 6]. These factors activated both specific and nonspecific immune cells, resulting in excessive inflammation. YKL-40 is a chitinase like protein that participates in the inflammation of tissue damage [7]. Current research on YKL-40 has focused on airway hyperresponsive diseases, autoimmune diseases, and pulmonary fibrosis [8], 9]. There are few studies on the relationship between YKL-40 and pneumonia.

IL-37, which belongs to the IL-1 family, is a novel inflammatory inhibitory factor with negative feedback regulation. It is closely related to tumors and inflammation in clinical practice [10]. At present, researches on IL-37 in patients with pneumonia focus on the relationship with novel coronavirus pneumonia [11]. However, there is almost no research on the relationship between *M. pneumoniae* pneumonia and IL-37.

Peripheral blood neutrophil to lymphocyte ratio (NLR) and platelet to lymphocyte ratio (PLR) are diagnostic indicators of inflammation, which can reflect the state of inflammatory response in the body. Moreover, NLR and PLR are computational indicators in blood routine analysis, which have the characteristics of low cost, simple and practical, and quick acquisition, and can be widely used in clinical work. NLR and PLR had also been demonstrated to predict the outcome of several types of infections, including CAP and COVID-19 [12], 13].

However, there has been no study on the relationship between MPP and the expressions of YLK-40, IL-37, NLR, and PLR. In present study, we detected the serum YKL-40 and IL-37 levels in MPP children, VP and healthy controls. We also compared the YKL-40 and IL-37 mRNA between all participants. Moreover, we investigated the correlation of YKL-40 and IL-37 levels with clinical parameters such as WBC, NLR, PLR, HsCRP and so on. We are attempting to confirm the relationship between these factors and MP. The present study aims to identify the differences between VP and MP and to develop a prediction tool for discriminating MPP from VP in children. In addition, we explored the critical cut-off values of the relevant biomarkers to find those that may be beneficial for the differential diagnosis of MPP and VP after hospital admission.

## Materials and methods

### Study population

The study was approved by the Ethics Committee of our hospital (code: [2024] KY005-01). Informed consent forms were signed by all subjects and/or their legal guardian(s). Children with pneumonia were admitted to the pediatric ward of our hospital from January to July 2024. The control group was children who underwent health checkups at our child health clinic during the same period. Due to the relevant regulations of our hospital, only children under 14 years old can be admitted for the medical treatment. Therefore, the population included in this study was less than 14 years old.

**MPP-Mycoplasma pneumoniae pneumonia**: The inclusion criteria were as below: (1) age1-13 years old; (2) Satisfy the diagnostic criteria of CAP; (3) Positive results for MPP polymerase chain reaction (PCR) tests of pharyngeal secretions.

**VP-Viral pneumonia:** The inclusion criteria were: (1) age1-13 years old; (2) Satisfy the diagnostic criteria of CAP; (3) Children diagnosed with influenza A, influenza B, adenovirus, rhinovirus, parainfluenza virus, COVID-19 and respiratory syncytial virus by quantitative real-time polymerase chain reaction (qRT-PCR) detection; (4) Macrolides and tetracycline antibiotics were not used before and after hospitalization.

**HC-Healthy control:** We also selected age- and sex-matched healthy children as the control group, who had no history of atopic diseases and no upper or lower airway diseases within 3 months. They underwent the same examinations as the pneumonia group.

**Exclusion criteria were:** 1) Failure to meet the inclusion criteria, or incomplete clinical data; 2) Presence of underlying diseases such as congenital heart disease, tuberculosis infection, metabolic diseases, connective tissue disease, inflammatory bowel disease, immunodeficiency diseases, HIV-positive, cerebral palsy, malignant tumors, bronchopulmonary dysplasia, or bronchiectasis; 3) Personal or family history of allergies, including atopic dermatitis, allergic rhinitis or asthma; 4) History of glucocorticoid, bronchodilator, immunosuppressant, antihistamine, or leukotriene receptor antagonist use within 4 weeks prior to hospital admission; 5) mixed infections of multiple pathogens; 6) mixed infections of bacteria.

**Sample size calculation:** The sample size was determined using PASS sample size software (2023).

### Methods

#### Pathogen detection

TaqMan qRT-PCR testing kit (NO.20213400256, Sansure Biotech INC.) was used to detect influenza A virus, influenza B virus, respiratory syncytial virus, adenovirus, rhinovirus, and *M. pneumoniae*. New coronavirus (2019-nCoV) antigen detection kit (NO.20203400064, Sansure Biotech INC.) was used to identify COVID-19. Similarly, we confirmed parainfluenza virus infection by fluorescence PCR method using parainfluenza virus types 1, 2 detection kits (NO.20243402033, Sansure Biotech INC.). Additionally, we used blood routine tests, procalcitonin, C-reactive protein, blood culture, sputum culture, PPD skin test, Chlamydia pneumoniae nucleic acid test, and *Bordetella pertussis* nucleic acid test to rule out bacterial infections, *Mycobacterium tuberculosis*, Chlamydia pneumoniae, and *B. pertussis* infections. For a small number of patients, bronchoalveolar lavage fluid and sputum Next-Generation Sequencing were used to exclude mixed infections.

#### Specimen collection

Blood samples from each participant were collected in the department within 24 h of admission. The blood samples were centrifuge at 3,000 rpm for 10 min, then the serum supernatants were collected. The serum samples were stored in a refrigerator at −80 °C before testing.

#### Data collection

The clinical data of pneumonia patients (including patient characteristics and laboratory data) came from their medical records. Laboratory data containing white blood cell (WBC), neutrophils (N), lymphocytes (L) and platelets (PLT) were recorded. NLR and PLR values were calculated from blood routine results.

#### Measurement of serum YKL-40 and IL-37 levels

We determined serum levels of YKL-40 and IL-37 using enzyme-linked immunosorbent assay (ELISA) kits (NO. JN20159 and JN19667, Shanghai Jining Industrial Co., Ltd.) and followed the manufacturer’s instructions. In the Excel worksheet, we created a linear regression curve of the standard using the standard concentration as the horizontal axis and the corresponding OD value as the vertical axis, and then used the curve equation to determine the concentration values for each sample. All tests were repeated. The YKL-40 results are expressed in units of ng/mL, while the IL-37 results are expressed in units of pg/mL.

#### Determination of YKL-40 mRNA and IL37 mRNA expression in peripheral blood mononuclear cells (PBMCs)

The mRNA expression levels of YKL-40 and IL-37 in PBMCs were directly analyzed by qRT-PCR (NO. 96a, Hangzhou Borui Technology Inc.). On the basis of the method provided by the manufacturer, TRIeasy™ LS Total RNA Extraction Reagent (NO. 19201ES60, Yeasen Biotechnology Co., Ltd.) was used to lyse peripheral blood mononuclear cells and RNA was extracted from the sample. Then RNA transcription and qRT-PCR detection were performed. Finally, relative expression was calculated using the 2^−ΔΔCq^ method [14].

### Statistical analysis

We used SPSS 19.0 statistical software package for analysis. The categorical variables were expressed in terms of numerical and percentage frequencies, and significant differences were evaluated using Pearson’s chi square test. Normality tests were performed on continuous variables. Normally distributed variables were expressed as mean±standard deviation (T±SD). T test or ANOVA was used to process these data. Skewness variables were represented as median and interquartile intervals (Q1, Q4). The significant differences between the median were evaluated using Mann-whitney u test or Kruskal Wallis test. We used Bonferroni correction for pairwise comparison among the three groups, and p<0.017 was considered significant. The relationship between variables were tested using the Spearman correlation test. The logistic regression analysis was conducted to identify significantly independent risk factors. The area under the curve (AUC) was calculated by drawing the receiver operating characteristic (ROC) curve and estimating its sensitivity and specificity. p values less than 0.05 was considered as a significant difference.

### Ethics statement

This study was approved by the Ethics Committee of The Second People’s Hospital of Changzhou, the Third Affiliated Hospital of Nanjing Medical University, China (approval no. [2024]KY005-01). Informed consent was obtained from all subjects and/or their legal guardian(s).

## Results

### Comparison of clinical characteristics at admission between the MPP group and the VP group

This study included 170 pneumonia patients, including 85 children with MPP (male/female ratio 44/41, mean age 7.04±2.41 years old) and 85 VP children (male/female ratio 53/32, mean age 6.34±2.58 years old). The statistical results showed that there was no significant difference in age and sex between the MPP group and the VP group (p>0.05). There was also no difference in the course of illness between the two groups at the time of enrollment. Consequently, the two groups were comparable. Compared with VP cases, MPP cases had a longer time of fever, hospitalization and glucocorticoid use, and required more bronchoscopy (all p<0.05). In addition, MPP children were more likely to be comorbid with plastic bronchiolitis. In terms of clinical symptoms, all children with pneumonia had coughing symptoms. Wheezing and gastrointestinal symptoms were more prevalent in VP cases than in MPP cases (p<0.05). However, the incidence of fever in MPP children was higher than that in VP children. We also evaluated the imaging findings and discovered that children with *M. pneumoniae* were more likely to have lung consolidation, whereas viral pneumonia was more likely to be of the bronchiolitis type (Table 1).

**Table 1:** The general characteristics and imaging findings of children with MPP and VP. The general characteristics and imaging findings of MPP and VP children were compared.

	MPP (n=85)	VP (n=85)	*T*(*Z*) *or χ* ^*2*^	p-Value
**General information**				
Sex (male/female)	44/41	53/32	*1.95*	*0.10*
Age, yr	7.04 ± 2.41	6.34 ± 2.58	*1.81*	*0.07*
Course of disease before recruitment, days	7.34 ± 2.84	6.88 ± 3.37	*0.96*	*0.34*
Fever duration, days	5.0 (4.0, 6.0)	4.0 (2.0, 6.0)	*−2.86*	*0.04*
Hospital stays, days	6.0 (6.0, 7.0)	6.0 (5.0, 7.0)	*−2.28*	*0.02*
Bronchoscopic use, n/%	21 (24.71 %)	3 (3.53 %)	*15.72*	*<0.001*
Glucocorticoid use, days	4.0 (2.0, 5.0)	1.0 (0, 3.0)	*−5.15*	*<0.001*
Combined plastic bronchitis, n/%	14 (16.47)	2 (2.35 %)	*9.94*	*0.002*
**Clinical presentation n, %**				
Fever	82 (96.47 %)	70 (82.35 %)	*6.92*	*0.02*
Cough	85 (100 %)	85 (100 %)		
Wheezing	5 (5.88 %)	19 (22.35 %)	*9.51*	*0.002*
Polypnea	3 (3.53 %)	4 (4.71 %)	*0.15*	*0.70*
Rash	4 (4.71 %)	2 (2.35 %)	*0.69*	*0.68*
Neurological Symptom	0	2 (2.35 %)	*2.02*	*0.16*
Gastrointestinal Symptom	8 (9.41 %)	19 (22.35 %)	*5.33*	*0.02*
**Imaging findings n, %**				
Pleural effusion	2 (2.35 %)	0	*2.02*	*0.50*
Lung consolidation	25 (29.41 %)	5 (5.88 %)	*16.19*	*<0.001*
Bronchiolitis	10 (11.76 %)	21 (24.71 %)	*4.77*	*0.03*

Italicized values represent test statistics (e.g., *t*, *χ* ^2^, *Z*) and their corresponding p-values. Non-italicized values are descriptive statistics (e.g., mean, standard deviation, counts, percentages).

### Demographic and inflammatory indicators of all participants

Demographic and inflammatory indicators of the study participants including MPP children, VP children and healthy controls were shown in Table 2. There was no significant difference in age and gender among these three groups (p>0.05). In addition, neutrophil, NLR, and PLR values in children with MPP were significantly higher than in the other two groups (p<0.05). Nevertheless, white blood cells levels in VP children were higher than that in MPP children and the control group (p<0.05). Comparing the MPP group and VP group, there were also significant differences in lymphocytes levels (p=0.02). No changes in other clinical variables (WBC and PLT) were found between the two groups. These findings were described in Table 2.

**Table 2:** Demographic and inflammatory indicators of all subjects. We compared the demographic and inflammatory indicators of all participants.

	MPP(group A) (n=85)	VP (group B) (n=85)	HC (group C) (n=85)	F(Z) or χ^2^	p-Value
Sex (male/female)	44/41	53/32	43/42	*2.88*	*0.24*
Age, yr	7.04 ± 2.41	6.34 ± 2.58	6.26 ± 2.70	*2.34*	*0.10*
WBC (10^9/L)	8.19 ± 2.90	8.23 ± 3.75	6.09 ± 1.46	*15.52*	*<0.001* ^b,c^
N (10^9/L)	4.99 ± 2.48	3.53 ± 2.04	2.99 ± 1.14	*23.46*	*<0.001* ^a,b^
L (10^9/L)	2.41 (1.86, 2.94)	2.86 (2.01, 5.67)	2.44 (2.08, 2.87)	*11.68*	*0.003* ^a,b,c^
PLT (10^9/L)	300.66 ± 93.04	289.95 ± 102.15	307.62 ± 59.20	*0.89*	*0.41*
NLR	1.94 (1.37, 2.64)	0.95 (0.48, 1.53)	1.16 (0.86, 1.49)	*51.49*	*<0.001* ^a,b,c^
PLR	129.94 ± 47.57	95.52 ± 50.05	128.96 ± 37.75	*15.81*	*<0.001* ^a,c^
YKL-40, ng/mL	150.57 ± 48.42	103.93 ± 38.72	85.78 ± 25.81	*63.15*	*<0.001* ^a,*b*,c^
IL-37, pg/mL	88.38 (33.96, 109.40)	45.06 (28.86, 87.51)	43.24 (28.67, 66.15)	*21.37*	*<0.001* ^a,b^
YKL-40 mRNA	4.24 (1.13, 15.84)	3.26 (1.59, 6.43)	1.75 (0.57, 3.76)	*21.76*	*<0.001* ^b*,*c^
IL-37 mRNA	6.75 (3.02, 14.56)	4.57 (1.90, 9.40)	1.59 (0.70, 4.47)	*48.68*	*<0.001* ^a*,*b,c^

Italicized values represent test statistics (e.g., *t*, *χ* ^2^, *Z*) and their corresponding p-values. Non-italicized values are descriptive statistics (e.g., mean, standard deviation, counts, percentages).

### Serum IL-37 and YKL-40 concentration in all study subjects

**Figure 1:**
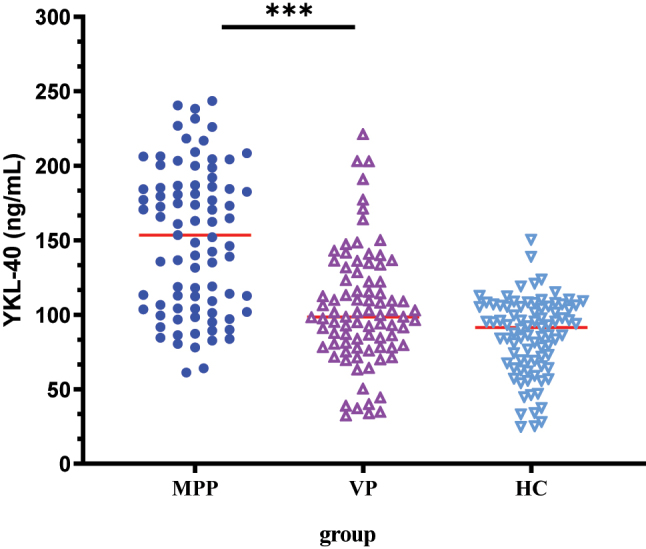
Serum YKL-40 level in all study subjects. Serum levels of YKL-40 were significant elevated in MPP patients as compared to VP and healthy controls.

**Figure 2:**
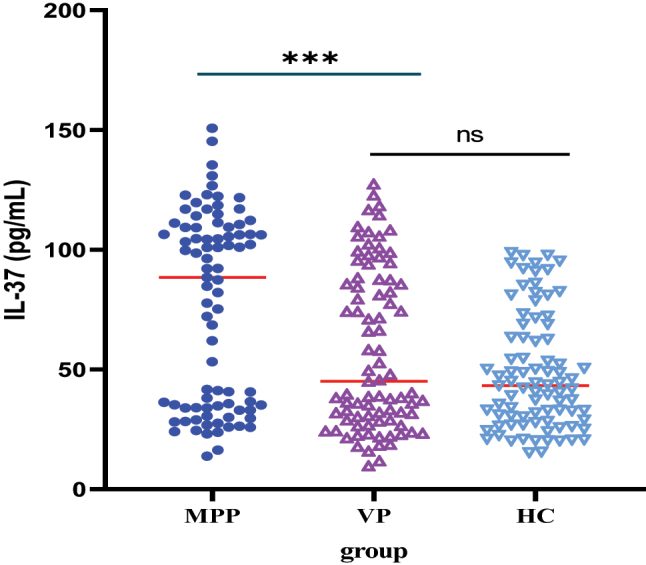
Serum IL-37 level in all study subjects. Children with MPP had the highest levels of serum IL-37.

ELISA results showed that serum YKL-40 and IL-37 levels were highest in children with MPP (Table 2, Figure 1, Figure 2). However, pairwise comparisons revealed no statistically significant difference in IL-37 level between the VP and control groups (Table 2).

### YKL-40 mRNA and IL-37 mRNA expression levels in patients with MP, VP and control group

**Figure 3:**
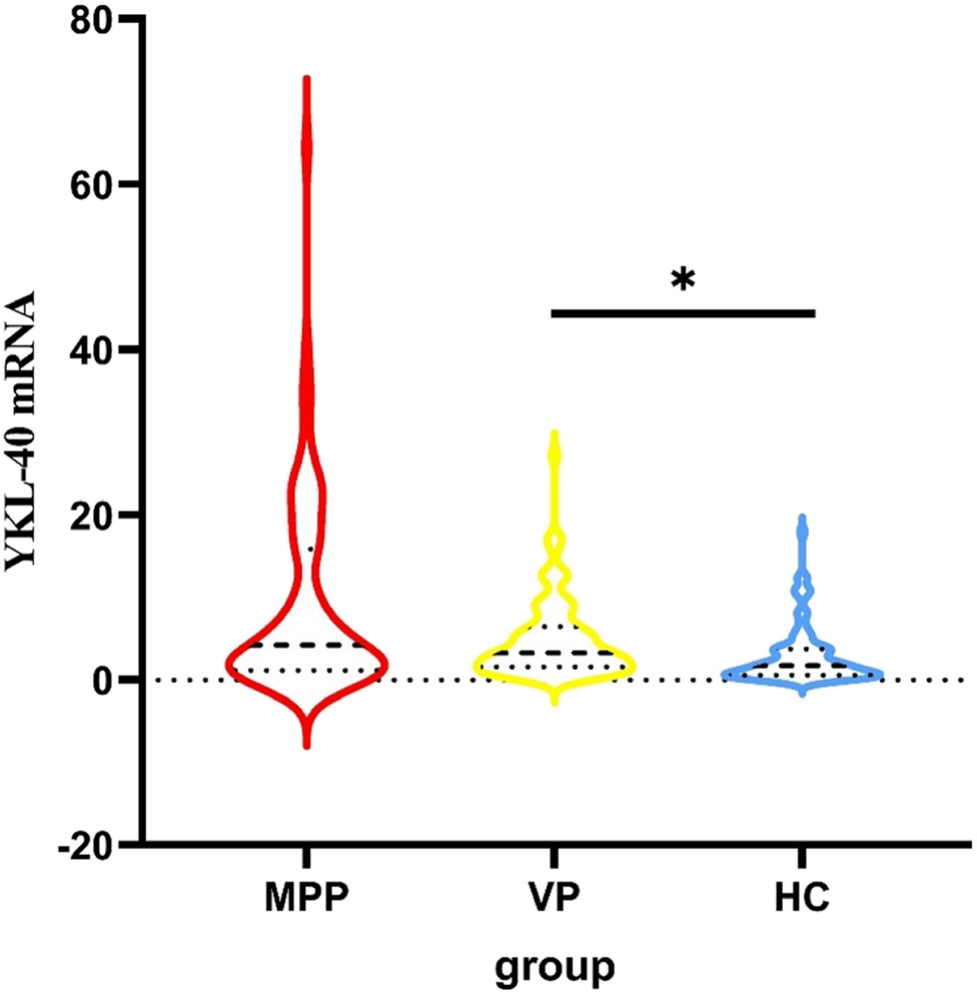
Serum YKL-40 mRNA expression in all groups. YKL-40 mRNA expressions in PBMCs were significantly increased in MPP compare to VP and healthy controls.

**Figure 4:**
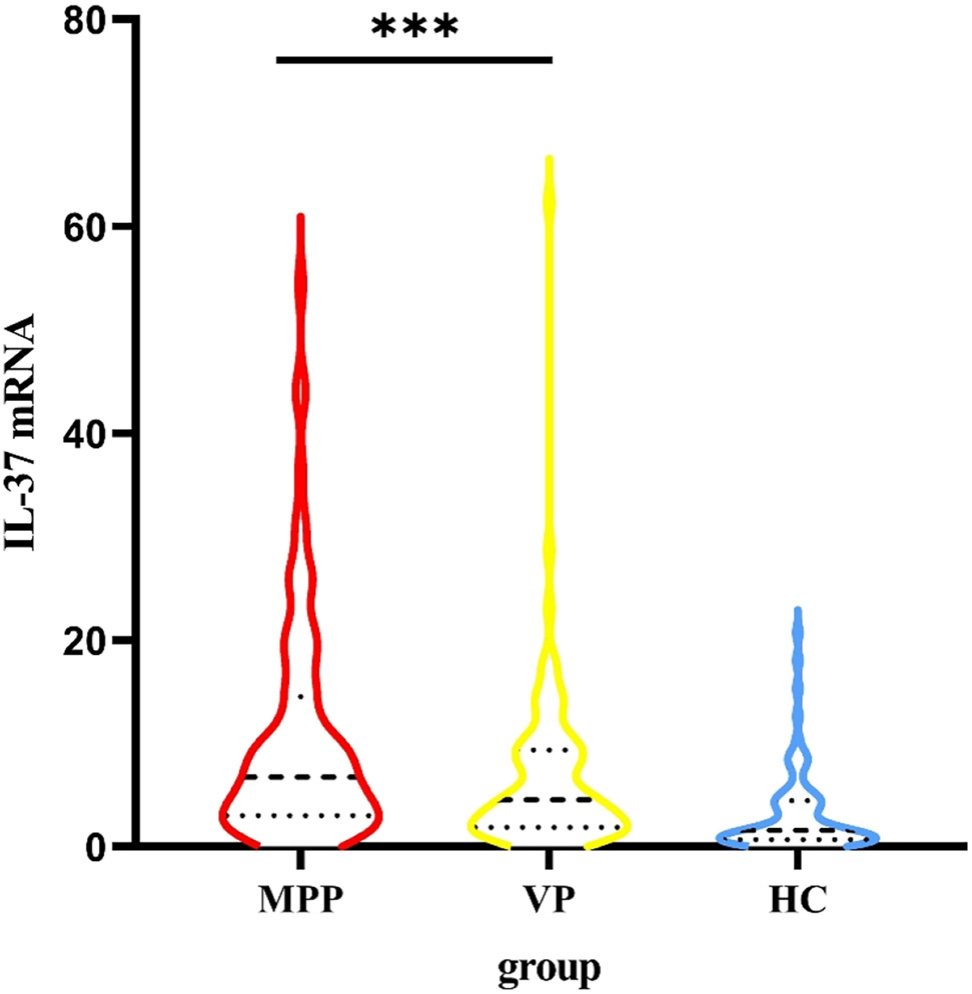
Serum IL-37 mRNA expression in all groups. MPP group had the highest YKL-40 mRNA expressions in PBMCs among the three groups.

The qRT-PCR data showed that MPP group had the highest YKL-40 mRNA and IL-37 mRNA expression in PBMCs among the three groups (Figure 3, Figure 4). In terms of pairwise comparison, there was no significant difference in YKL-40 mRNA expression levels between the MPP group and the VP group (Table 2).

### Correlations between serum YKL-40, IL-37 and relevant clinical indicators in MPP group

**Figure 5:**
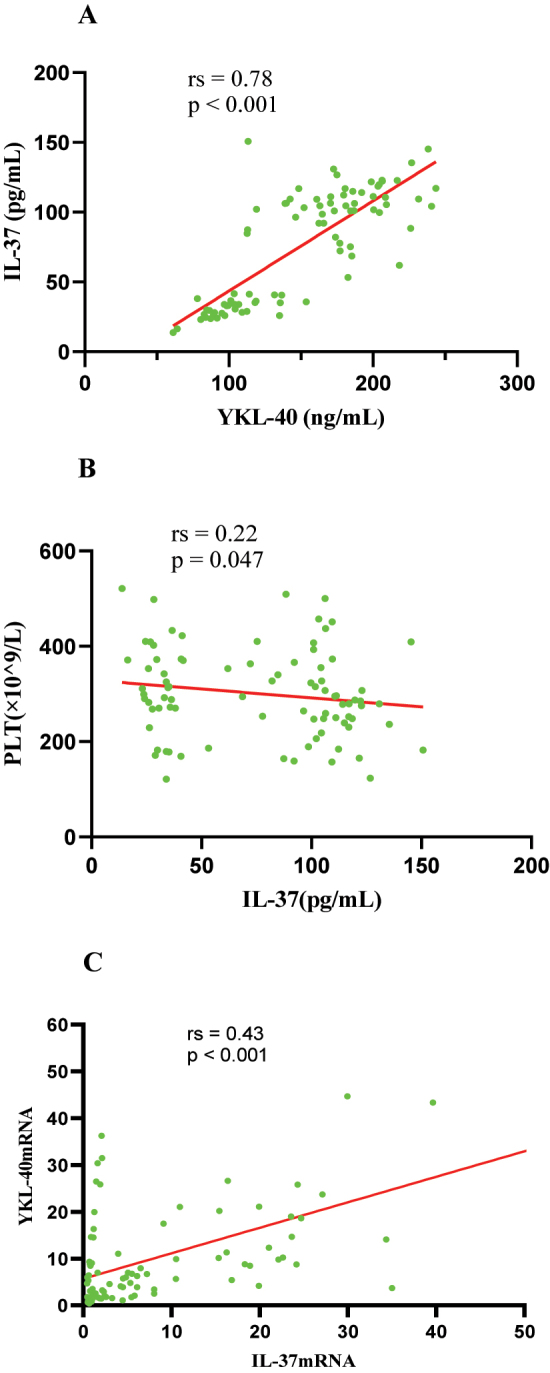
Correlations between the inflammatory factors. (Figure 5A) Spearman correlation test showed that serum IL-37 was positively correlated with YKL-40. (Figure 5B) Serum IL-37 was negatively correlated with PLT. (Figure 5C) Serum IL-37 mRNA was positively correlated with YKL-40 mRNA.

In MPP group, the correlation between serum YKL-40, IL-37 levels and clinical features was then examined. The results showed a positive correlation between IL-37 and YKL-40 (rs=0.78, p<0.001) (Figure 5a) and a negative correlation with PLT (r=−0.22, p=0.047) (Figure 5b) in MPP children. However, Serum YKL-40 and IL-37 levels were not correlated with other clinical indicators (WBC, N, NLR, PLR and HSCRP). In addition, we conducted statistical analysis on the correlation between the expression of IL-37 mRNA and YKL-40 mRNA and other clinical indicators. IL-37 mRNA was found to be correlated with YKL-40 mRNA (rs=0.43, p<0.001) (Figure 5C).

### Reliability of clinical and laboratory indicators in identifying MPP

We used logistic regression analyses to assess the ability of clinical indicators and laboratory parameters to identify MP patients in pneumonia patients. Comparing MPP and VP by multivariate logistic regression analysis, lung consolidation, bronchiolitis, NLR and YKL-40 were independent predictors associated with MPP (Table 3).

**Table 3:** The independent predictors of MP infection in CAP patients. The logistic regression analyses of factors were used for the prediction of MP. Lung consolidation, bronchiolitis, NLR and YKL-40 were independent predictors associated with MPP.

Variable	*β*	SE	Wald	p-Value	OR	95 % CI	
Fever	0.32	0.95	0.12	0.72	1.40	0.22	8.89
Wheezing	−0.87	0.67	1.68	0.20	0.42	0.11	1.56
Gastrointestinal symptom	−1.20	0.63	3.57	0.06	0.30	0.09	1.05
Lung consolidation	1.34	0.63	4.49	0.03	3.83	1.11	13.29
Bronchiolitis	−1.31	0.59	4.88	0.03	0.27	0.08	0.86
NLR	0.51	0.22	5.35	0.02	1.67	1.08	2.58
PLR	0.01	0.005	0.85	0.36	1.01	0.99	1.01
YKL-40	0.03	0.008	15.79	<0.001	1.03	1.02	1.05
IL-37	−0.01	0.01	1.61	0.20	0.99	0.99	1.09
IL-37 mRNA	0.04	0.02	2.33	0.13	1.04	0.64	8.86

### The discriminatory power of YKL-40 and NLR for MP vs. VP

**Figure 6:**
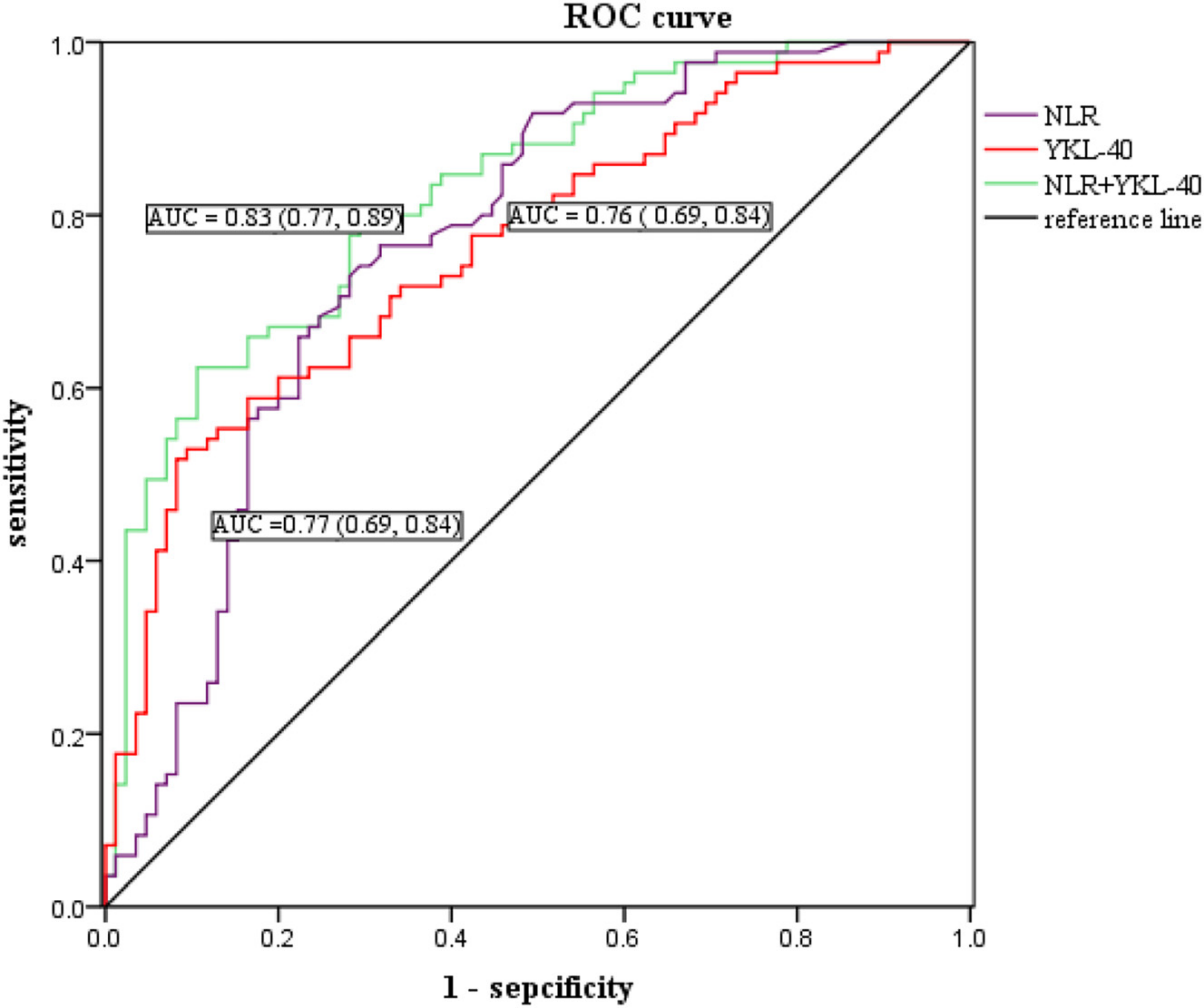
The ROC curve of each biomarker to identify MPP against VP. The combined metrics (NLR + YKL-40) had the highest area values and high sensitivity and specificity.

The ROC curves for two indicators (NLR and YKL-40) in patients with MPP and VP group were generated. The AUCs of NLR, YKL-40 and the combined indicator (NLR+YKL-40) for MPP patients and VP patients were 0.77, 0.76 and 0.83, respectively (Table 4). Based on the ROC curve analysis, the optimal thresholds for NLR and YKL-40 were 1.44 and 148.37 ng/mL, respectively. The combined metrics had the highest area values and high sensitivity and specificity (Figure 6).

**Table 4:** Discriminatory value of independent correlation factors for MPP. The AUC and the optimal cut-off points with predictive biomarkers for MPP were shown.

Independent factors	AUC	Cut-off	95%CI	Sensitivity	Specificity
NLR	0.77	1.44	0.69 0.84	0.73	0.72
YKL-40	0.76	148.37	0.69 0.84	0.53	0.91
NLR+YKL-40	0.83		0.77 0.89	0.62	0.89

## Discussion

MP and viruses are important pathogens that cause pulmonary infections, and they have similar clinical symptoms. Both MP and viruses can cause greater immune system damage to the infected organism [15]. When the pathogen cannot be identified, combination therapy is commonly used. Traditional combination therapy can lead to more side effects. Currently, there are few studies that focus on the differences between VP and MPP [16]. The distinction between MP and viral pneumonia has remained problematic. It is possible to delay early diagnosis and guided treatment, which is detrimental to disease regression. It is necessary for us to enhance clinicians’ awareness of the clinical differences between MPP and VP in children, thereby facilitating timely clinical decisions.

In terms of demographic characteristics, compared with VP cases, MPP cases had longer fever duration, longer hospitalization time, and higher rates of glucocorticoid use and bronchoscopy. In addition, we found that children with MPP were more probably to have a combination of plastic bronchitis. This result may be associated with an overactive immune response in MP children [17]. In this study, VP children were more likely to experience wheezing and gastrointestinal symptoms than those with MPP. It’s worth noting that fever was a characteristic symptom of MPP, with significantly higher rates when compared to VP. We also evaluated the imaging findings and discovered that MPP children were more likely to have lung consolidation, whereas VP was more likely to be of the bronchiolitis type. This was consistent with a previous finding [18]. This may be due to the fact that MP easily damages the alveolar wall directly, causing the accumulation of exudate in the alveoli and forming consolidation. Additionally, the virus mostly infiltrates the bronchioles, resulting in lumen constriction and obstruction.

Due to the time-consuming isolation of pathogenic microorganisms, which cannot be used for early disease diagnosis, other biomarkers are used in clinical practice to distinguish viral infections from mycoplasma infections. Based on previous studies, we selected four common biological indicators (NLR, PLR, YKL-40 and IL-37) as our research subjects. The NLR and PLR are recognized as simple, rapid, and widely available indicators of systemic inflammation and infection [19]. A study had found that NLR had diagnostic value in differentiating between bacterial and viral pneumonia [20]. Besides, another research performed by Dan Li et al. confirmed that NLR can predict poor prognosis in MPP patients [21]. However, it had been shown that PLR reflects the degree of platelet activation, which has been associated with infectious diseases, blood disorders and immune disorders [22]. Few reports demonstrated that the NLR or PLR may contribute to the differential diagnosis of MPP. According to our statistics, the NLR can predict MP with an AUC of 0.77, if a cutoff>1.44 was used. Remarkably, the PLR failed to distinguish between MP and VP. This result may be due to the lack of significant differences in platelet activation between MPP and VP.

YKL-40 is a new marker for inflammation and remodeling. Numerous researches have indicated that YKL-40 may be closely related to inflammatory responses [23]. Patients with more severe pneumonia had greater YKL-40 levels [24]. Yang XG et al. suggested that the prognosis of severe viral pneumonia is correlated with YKL-40 levels [25]. But the study about YKL-40 in distinguishing MPP from VP is still infrequent. It is necessary to further clarify the reliability of YKL-40 as a MP diagnostic marker. We have found that serum YKL-40 levels were especially higher in MPP children compared with VP children. Due to the relationship between YKL-40 and the severity of the inflammatory response, these higher levels indicate that pneumonia caused by MP infection may be more severe compared to viral infection.

YKL-40 mRNA and YKL-40 are different molecular forms in the same gene expression process. The former is the gene transcription product encoding chitinase-like protein 40, while the latter is the protein product translated from this gene. They have synergistic effects in certain diseases. A previous study showed that compared with healthy non-smokers, chronic obstructive pulmonary disease (COPD) patients showed elevated serum concentrations of YKL-40 and increased YKL-40 mRNA expression in PBMCs [26]. Unfortunately, although YKL-40 expression levels in PBMCs were higher in MPP children than VP children, it was no significant difference. This result may be due to technical factors (such as measurement mistakes, sample preservation and handling) or biological factors (such as post-transcriptional regulation) [27].

IL-37 is known to inhibit innate immune responses and regulate acquired immune responses [28]. Previous studies have also shown that IL-37 levels are elevated in many inflammatory diseases, such as atopic dermatitis, SARS-CoV-2 infection and autoimmune diseases [29], [30], [31]. However, there was no study on the relationship between IL-37 and MP. In the present study, we found that serum IL-37 levels have been elevated in patients with pneumonia compared to healthy controls, which is inconsistent with the research by Wang JL et al. [32]. The inconsistency of this finding may be explained with the large age gap in the study population and the undifferentiated pathogens in CAP patients. Besides, serum IL-37 levels were higher in MPP patients than in VP patients and control cases. Regarding IL-37 mRNA expression, MPP patients exhibited significantly elevated expression levels of IL-37 mRNA in PMBCs. These results reveal that IL-37 may be associated with MP infection. However, Logistic regression analysis showed that IL-37 was not a risk factor for MPP. Due to the lack of other studies on the correlation between IL-37 and MPP, the underlying mechanisms remain unclear. It is uncertain whether this is related to the limited sample size in our study. Further research with increased sample size is needed to confirm the correlation.

Furthermore, this study recorded significant positively correlations between serum YKL40 levels and IL-37 levels, as well as YKL-40 mRNA expression levels and IL-37 mRNA expression levels in MPP children. This may be due to the regulation of YKL-40 expression by multiple cytokines. Previous studies had found that YKL-40 expression was regulated by some inflammatory factors, such as IL-6, IL-13, IL-1 and interferon-γ [33]. YKL-40 increased the production of pro-inflammatory cytokine (IL-8) in bronchial epithelial cells by activating MAPK and NF-kB pathways [34]. But.

IL-37 forms a complex with IL-18Rα and IL-1R8, transmitting anti-inflammatory signals by inhibiting the MAPK and NF-κB pathways [35]. This indicates that they may be involved in the development and progression of diseases through complex interactions. Definitely, there is currently almost no research on the relationship between YKL-40 and IL-37, and further researches are needed to explore the mechanisms of their correlation.

Through multivariable logistic regression analysis, we found that NLR, YKL-40 and lung consolidation were independent risk factors for MP. The combined indicator (YKL-40 and NLR) predicted MPP as compared to VP with an area under the ROC curve of 0.83. To our knowledge, the potential of NLR and YKL-40 in distinguishing MPP from VP has never been confirmed. As a result, it was proposed that children with aberrant expressions of YKL-40 and higher levels of NLR be included as priority screening subjects for MPP in CAP. Further research is required to verify our outcomes.

Nevertheless, there are some limitations in this study. First, the molecular mechanisms between YKL-40, IL-37 and MPP are still unclear, and further researches on the molecular mechanisms of YKL-40 and IL-37 are needed. Second, we did not test the performance of YKL-40 and NLR in patients with severe mycoplasma pneumonia, refractory mycoplasma pneumonia, and mild mycoplasma pneumonia. In the future, our team will continue to conduct the research in this area. Third, we have tried to exclude patients with mixed infections as much as possible. However, due to limitations in laboratory testing, it is not possible to completely rule out the possibility of co-infection with bacteria. Fourth, the AUC value of the combined indicator (NLR+YKL-40) is 0.83, with high specificity but relatively low sensitivity, which needs to be validated through subsequent large-scale experiments. Finally, our study was a single-center study with a limited number of subjects, and the experimental results have some limitations that need to be confirmed by subsequent large-scale multicenter studies. We will conduct multicenter studies in the future to validate the clinical applicability of our research.

## Conclusions

In conclusion, we conducted a comparative study on clinical and laboratory indicators of MPP and VP in children. We found that NLR, YKL-40, and lung consolidation are independent risk factors for MPP in children. We calculated NLR cutoff of 1.44 and YKL-40 cutoff of 148.37 ng/mL, which can be used to distinguish between MPP and VP. The combined use of these indicators (NLR and YKL-40) has the highest discriminatory value. We hope to improve the understanding of clinical differentiation between MPP and VP in children, and help clinicians to make timely and correct clinical treatments.
